# BCC Structural Stability and Deformation Behavior of Ti-Mo Alloys: A Cluster-Model-Embedded First-Principles Study

**DOI:** 10.3390/ma19153159

**Published:** 2026-07-23

**Authors:** Xiaoyun Li, Qixiang Zhang, Yulong Zhao, Ben Niu, Chaoli Ma, Wenlong Xiao, Qing Wang

**Affiliations:** 1 School of Materials Science and Engineering, Key Laboratory of Materials Modification by Laser, Ion and Electron Beams (Ministry of Education), Dalian University of Technology, Dalian 116024, China; lixiaoyun@mail.dlut.edu.cn (X.L.); zhangqixiang@mail.dlut.edu.cn (Q.Z.); zhao_yuloong@163.com (Y.Z.); asualywy@163.com (B.N.); 2 Tianmushan Laboratory, Hangzhou 311115, China; machaoli@buaa.edu.cn (C.M.); wlxiao@buaa.edu.cn (W.X.); 3 School of Materials Science and Engineering, Beihang University, Beijing 100083, China

**Keywords:** Ti-Mo alloys, cluster-formula approach, first-principles calculations, deformation behavior, BCC structural stability

## Abstract

**Highlights:**

- The β-phase stability and deformation behaviors of Ti-Mo alloys are investigated.
- Increasing Mo enhances the β-phase stability of Ti-Mo alloys.
- The negative *G* of Ti-4.7Mo alloy indicates mechanical instability of β phase.
- *G* serves as a valid descriptor linking β-phase stability to deformation behavior.

**Abstract:**

To elucidate the intrinsic link between the body-centered cubic (BCC) structural stability and the deformation mechanisms of Ti-Mo alloys, first-principles (FP) calculations based on a cluster model are performed for Ti-*x*Mo (*x* = 2.3, 4.7, 7.8, and 12.5 at. %) binary alloys. Structural models are constructed by embedding a cluster unit within a 4 × 4 × 4 BCC supercell. The formation energies (*E_f_*) and binding energies (*E_b_*) of the α and β phases indicate that BCC structural stability increases with higher Mo content, and that the Ti-12.5Mo alloy is energetically favored to form a single β phase. For the metastable Ti-4.7Mo alloy, the negative value of $G_{\left(101)[10\bar{1}\right]}$ indicates lattice softening, while the value of *G*_(101)[010]_ = 3.9 GPa is lower than those of $G_{(323)[1\bar{3}1]}$ and $G_{(110)[1\bar{1}1]}$. Both features facilitate the stress-induced α″ transformation. Conversely, in the stable Ti-12.5Mo alloy, *G*_(011)[100]_ reaches 36 GPa, substantially exceeding $G_{\left(123)[11\bar{1}\right]}$, thereby suppressing α″-phase transformation and promoting dislocation slip. The present cluster model-embedded first-principles calculations demonstrate that the shear modulus serves as a valid descriptor linking BCC structural stability to deformation behavior in Ti-Mo alloys.

## 1. Introduction

Ti-Mo alloys are widely employed in biomedical and chemical engineering due to their high specific strength, excellent corrosion resistance, and favorable biocompatibility [1,2,3]. Pure Ti adopts a hexagonal close-packed (HCP) α phase at low temperatures and a body-centered cubic (BCC) β phase at high temperatures. Mo, as a β-stabilizing element, lowers the martensite start temperature (*M_s_*) [4,5,6], allowing the β phase to be retained at room temperature in a metastable or stable state. At low Mo contents, insufficient β stability leads to the formation of metastable phases such as α′, α″, and ω upon cooling. The β phase becomes progressively more stable with increasing Mo content. Notably, the properties of these alloys are intimately linked to the stability of the BCC structure. When the BCC structure is metastable, the alloy may undergo stress-induced α″ martensitic transformation, {332}<113>_β_ twinning, and {112}<111>_β_ twinning during deformation [6,7,8]. Typically, the α″-phase transformation that occurs in the elastic stage leads to a double-yielding phenomenon and reduces the yield strength (*σ*_YS_). For example, the *σ*_YS_ of β-metastable Ti-6.4Mo (at. %) alloy is below 500 MPa [9], which is lower than that of β-stable Ti-17.6Mo (at. %) alloy (*σ*_YS_ = 798 MPa [10]). In the plastic stage, transformation-induced plasticity (TRIP), twinning-induced plasticity (TWIP) and dynamic Hall–Petch effects endow the alloy with excellent ductility and strain hardening capacity [11,12,13,14]. For instance, the β-metastable Ti-8.1Mo (at. %) alloy achieves a total elongation of about 40% due to the TWIP effect from stress-induced {332}<113>_β_ twinning [15], exceeding conventional TC4 alloy (*ε* = 11% [16]) and β-stable Ti-17.6Mo (at. %) alloy (*ε* = 17% [10]). Thus, stress-induced phase transformation and twinning significantly influence the mechanical properties of Ti-Mo alloys.

Although experimental studies have established that Mo addition stabilizes the β phase and shifts the deformation mechanisms from stress-induced α″ martensitic transformation and twinning to dislocation slip [16,17,18], the intrinsic mechanisms governing these processes warrant further exploration. First-principles (FP) calculations based on density functional theory (DFT) have been employed to investigate phase stability and transformation behavior in the Ti-Mo binary system [19,20,21,22]. For example, using a β-phase supercell model constructed from “-Mo-Ti-Mo-” linear units or their combinations, previous studies demonstrated that β-phase stability increases with Mo content and that *C*′ softening results in a low Young’s modulus of the β phase [20]. With the same supercell model, the formation of α″ martensite and the ω phase was also examined. The results indicated that the formation of the α″ martensite at low Mo content is governed by the softening effect of *C*′ and *E*_100_, while the formation of the ω phase relates to the softening of *G*_111_ [22]. Nevertheless, modeling metastable alloys often relies on a special quasi-random structure (SQS) or the above supercell model, requiring selection of an optimal configuration that reflects the interatomic interactions. In solid solutions, these interactions are characterized by chemical short-range orders (CSROs) [23,24]. In fact, solid-solution properties depend on short-range ordered structures. An FP study of Al-TM (TM = Ti, Zr, Hf) alloys demonstrated that configurations incorporating CSROs produce formation energies in much better agreement with calorimetry data than random structures [25]. Therefore, properly incorporating CSROs into solid-solution alloy models is essential for achieving accurate computational predictions.

Based on the concept of CSROs, the cluster-plus-glue-atom model was proposed in the previous work [26,27,28], which comprises a solute-centered nearest-neighbor polyhedron (cluster) and glue atoms in the interstices between clusters. This model captures both the strong interaction (Δ*H* < 0) between solute atoms at the cluster center and the base solvent atoms, as well as the weak interaction (Δ*H* > 0) between glue atoms and the base, defining a structural unit [cluster](glue atom)*_m_* (*m* is the number of glue atoms). In a previous study on the Al_2_M_14_ (M = Ni_1_Co_1_Fe_2_Cr_1_) alloy, the cluster model was validated by neutron diffraction. The simulated pair-distribution function (PDF) from the cluster model agrees well with the measured PDF within the first two peaks, demonstrating that the cluster model accurately describes the chemical short-range order in the solid solution [28]. This model provides an effective and accurate structural input for FP calculations and has been successfully applied to various alloys. For instance, the cluster formula was used to design a series of Ti-Zr-Nb-Hf high-entropy alloys and construct cluster models as inputs for FP calculations. Among them, the [Ti-Zr_8_Hf_4_Ti_2_]Nb_3_ alloy with BCC structure exhibited the lowest formation energy (7.82 kJ/mol) and was experimentally verified to form a single-phase BCC solid solution, matching computational results [29]. In this study, the structural models of Ti-*x*Mo alloys (*x* = 2.3, 4.7, 7.8, and 12.5 at. %) are constructed using the cluster model for FP calculations. The formation energies (*E_f_*) and binding energies (*E_b_*) of the α and β phases are calculated to link Mo content with BCC structural stability. Subsequently, the Ti-4.7Mo and Ti-12.5Mo alloys are selected to evaluate mechanical stability. Finally, considering the β ⟶ α″ transformation, {332}<113>_β_ twinning, and dislocation slip, the orientation-dependent shear moduli of the Ti-4.7Mo and Ti-12.5Mo alloys are calculated to clarify the intrinsic relationship between structural stability and deformation behavior in Ti-Mo alloys.

## 2. First-Principles Calculations

### 2.1. Construction of Cluster Structural Models

In the cluster-plus-glue-atom model for BCC solid solution alloys, the nearest-neighbor cluster is typically a rhombic dodecahedron with a coordination number of 14, consisting of 8 atoms in the first-neighbor shell and 6 atoms in the second-neighbor shell. According to Friedel oscillation theory [27,30], the number of glue atoms is ideally determined as *m* = 1, resulting in a total of 16 atoms per ideal cluster unit. In the present Ti-Mo binary system, the cluster formula can be expressed as [(Ti,Mo)-Ti_14_](Ti,Mo) with *Z* = 16. This assignment is supported by the strong interaction between Ti and Mo (Δ*H*_Ti-Mo_ = −4 kJ·mol^−1^). The occupation of Mo atoms depends on their concentration. In Mo-deficient alloys, Mo preferentially occupies the cluster center, while the remaining cluster centers are occupied by Ti; once the center site is filled, the excess Mo then occupies the glue sites.

**Figure 1:**
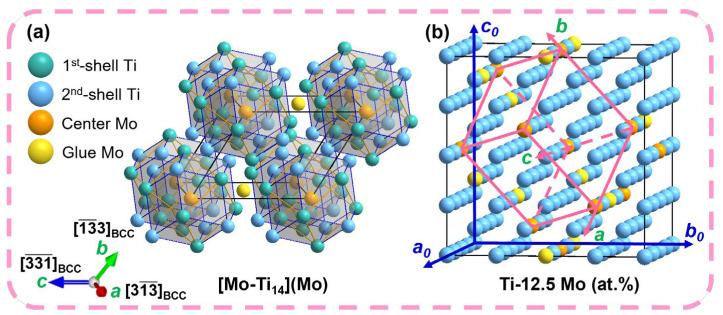
(**a**) Cluster unit of [Mo-Ti_14_](Mo), with atoms colored as follows: 1st-shell Ti (green), 2nd-shell Ti (blue), center Mo (orange), and glue Mo (yellow). (**b**) Structural model of Ti-12.5Mo alloy for FP calculations.

Employing the formula [(Ti,Mo)-Ti_14_](Ti,Mo), a series of Ti-Mo binary alloys are designed: [(Ti_0.625_Mo_0.375_)-Ti_14_](Ti) (2.34 at. % Mo), [(Ti_0.25_Mo_0.75_)-Ti_14_](Ti) (4.69 at. % Mo), [Mo-Ti_14_](Ti_0.75_Mo_0.25_) (7.81 at. % Mo), and [Mo-Ti_14_](Mo) (12.50 at. % Mo). Their corresponding cluster units, each containing 16 atoms, were constructed. The basis vectors *a*, *b*, and *c* of these cluster units correspond to $[3\bar{13}]$, $[\bar{13}3]$, and $\left[\bar{331}\right]$ of the basis BCC lattice, respectively. The lattice parameters are *a* = *b* = *c* = $\sqrt{19}a_{0}/2$, *α* = 118.27°, *β* = 99.09°, and *γ* = 61.73°, where *a*_0_ is the lattice constant of the basis BCC unit. The cluster unit [Mo-Ti_14_](Mo) is shown in Figure 1a, with atoms colored as follows: 1st-shell Ti (green), 2nd-shell Ti (blue), center Mo (orange), and glue Mo (yellow). Atomic coordinates are provided in Table A1.

**Figure A1:**
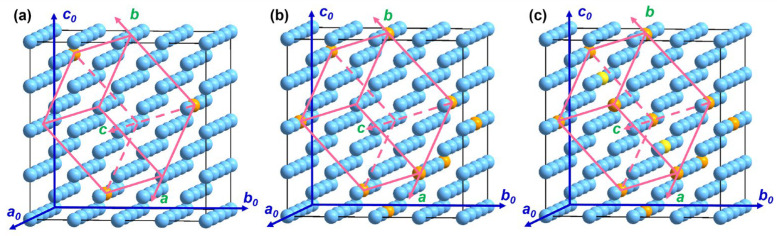
Structural model of (**a**) Ti-2.3Mo, (**b**) Ti-4.7Mo, and (**c**) Ti-7.8Mo (at. %) alloys for FP calculations, with atoms colored as follows: Ti (blue), center Mo (orange), and glue Mo (yellow).

**Figure A2:**
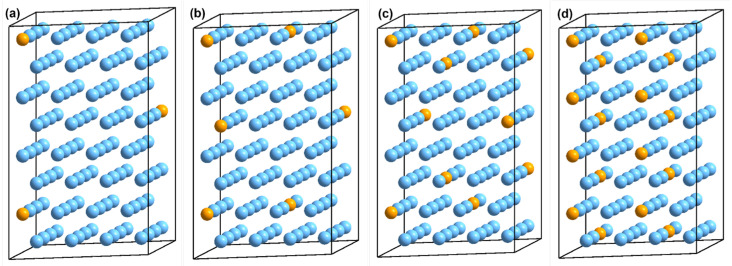
α-phase model of (**a**) Ti-2.3Mo, (**b**) Ti-4.7Mo, (**c**) Ti-7.8Mo, and (**d**) Ti-12.5Mo (at. %) alloys for FP calculations, with atoms colored as follows: Ti (blue), Mo (orange).

Because the cluster unit has a triclinic lattice whereas deformation analysis uses BCC orientations, each cluster unit was embedded in a 4 × 4 × 4 BCC supercell containing 128 atoms to facilitate shear moduli calculations and deformation analysis, with 3, 6, 10 and 16 Mo atoms placed within the supercell. Accordingly, the structural models of Ti-*x*Mo (*x* = 2.3, 4.7, 7.8, and 12.5 at. %) were constructed, exhibiting monoclinic symmetry due to Mo occupation. Figure 1b illustrates the structural model of Ti-12.5Mo alloy ([Mo-Ti_14_]Mo). Other models are detailed in Figure A1, and the coordinates of Mo atoms are detailed in Table A2. In addition, the constructed α phase models are shown in Figure A2. The structure models are provided as Model S1 (Supplementary models.Zip).

### 2.2. Calculation Methods

**Figure A3:**
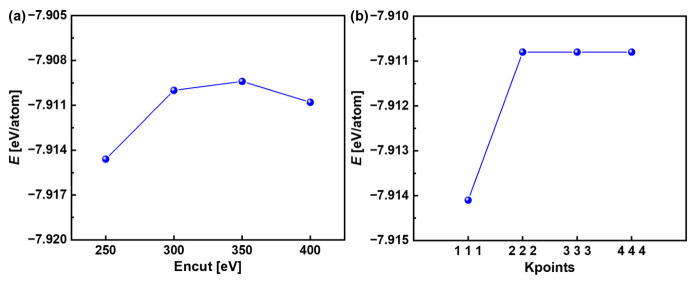
Convergence of the total energy *E* with respect to (**a**) the cutoff energy Encut and (**b**) the k-point mesh for Ti–4.7Mo.

FP calculations were performed using the Vienna Ab initio Simulation Package (VASP) [31,32,33,34]. The electronic exchange–correlation potential was treated within the generalized gradient approximation (GGA) using the Perdew–Burke–Ernzerhof (PBE) formulation [35]. The projector-augmented-wave (PAW) method was employed to describe the core electrons [36,37], with valence electron configurations set as 3p^6^3d^3^4s^1^ for Ti and 4p^6^4d^5^5s^1^ for Mo. The plane-wave cutoff energy was set to 400 eV, corresponding to the VASP default PREC = Normal setting. With this setup, the automatically generated real-space FFT grids were NGX × NGY × NGZ = 70 × 70 × 70 for the coarse grid and NGXF × NGYF × NGZF = 140 × 140 × 140 for the fine grid. The energy convergence criteria were set to 1 × 10^−8^ eV for electronic relaxation and 1 × 10^−7^ eV/atom for ionic relaxation. The Brillouin zone integration was performed using the Monkhorst–Pack scheme [38]. A 3 × 3 × 3 k-point mesh was used for the β-phase supercell and a 3 × 3 × 2 mesh for the α-phase supercell, ensuring an accuracy of 2 meV/atom (Figure A3). The density of states (DOS) calculation was performed using a 4 × 4 × 4 k-point mesh. All structural models were fully relaxed to reach their lowest-energy equilibrium configurations. The elastic constants were calculated using the optimized high-efficiency strain-matrix sets (OHESS) approach implemented in the ElasTool package [39]. Deformations of the crystal lattice were introduced via ElasTool’s automated routine with four strain values: −0.01, −0.005, 0.005, and 0.01. For the monoclinic crystal system, 13 independent elastic constants (*C*_11_, *C*_22_, *C*_33_, *C*_44_, *C*_55_, *C*_66_, *C*_12_, *C*_13_, *C*_23_, *C*_15_, *C*_25_, *C*_35_, *C*_46_) were determined. The polycrystalline Young’s modulus *E* was derived using the Voigt–Reuss–Hill (VRH) method [40], according to Equations (1)–(3):

(1) $$E=\frac{9G_{VRH}B_{VRH}}{G_{VRH}+B_{VRH}}$$

(2) $$G_{VRH}=\frac{G_{V}+G_{R}}{2}$$

(3) $$B_{VRH}=\frac{B_{V}+B_{R}}{2}$$

where *G_V_*, *G_R_*, and *G_VRH_* represent the shear moduli from Voigt, Reuss, and VRH methods, respectively. *B_V_*, *B_R_*, and *B_VRH_* denote the bulk moduli from Voigt, Reuss, and VRH methods. The shear moduli (*G_V_*, *G_R_*) and bulk moduli (*B_V_*, *B_R_*) were calculated from elastic constants, as detailed in Appendix A (Note A1).

The deformation behavior is related to the BCC structural stability, which can be evaluated by comparing the formation energy (*E_f_*) and binding energy (*E_b_*) of the α and β phases. The *E_f_* and *E_b_* are given by Equations (4) and (5) [26,41,42]:

(4) $$E_{f}=\frac{E_{tot}-\sum E_{M}}{N}$$

(5) $$E_{b}=\frac{E_{tot}-\sum E_{single}}{N}$$

where *E_f_* represents the energy required to form the alloy, defined as the difference between the total energy of the optimized structure (*E_tot_*) and the sum of the energies of individual atoms in their stable elemental structures (*E_M_*, HCP for Ti and BCC for Mo), while *E_b_* reflects the energy associated with atomic bonding relative to isolated states (*E_single_*). *N* is the total number of atoms in the model.

## 3. Results and Discussion

### 3.1. Formation and Binding Energies of Ti-Mo Binary Alloy

**Figure 2:**
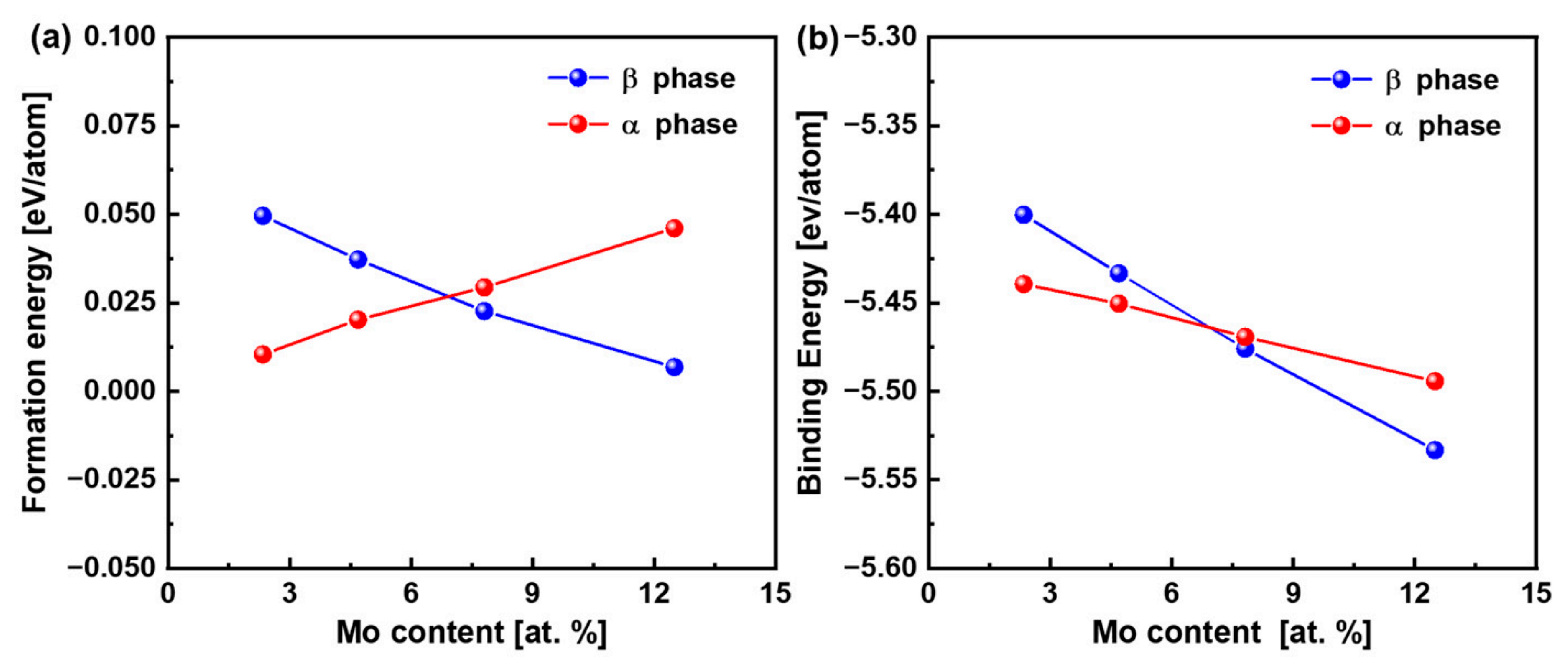
Variation of (**a**) formation energy (*E_f_*) and (**b**) binding energy (*E_b_*) with Mo content for α and β phases in Ti-*x*Mo (*x* = 2.3, 4.7, 7.8, 12.5 at. %) alloys.

To evaluate the BCC structural stability of Ti-Mo binary alloys, the formation energies *E_f_* and binding energies *E_b_* of the α (HCP) and β (BCC) phases were calculated, as shown in Figure 2. The β phase in the Ti-2.3Mo alloy exhibits the highest value of *E_f_* = 0.050 eV/atom, while the α phase shows the lowest value of *E_f_* = 0.009 eV/atom, indicating the least stable BCC structure. As the Mo content increases, the *E_f_* of the β phase decreases from 0.037 eV/atom in Ti-4.7Mo to 0.023 eV/atom in Ti-7.8Mo, and further to 0.007 eV/atom in Ti-12.5Mo alloy (Figure 2a). In contrast, the *E_f_* of the α phase increases with Mo content, being *E_f-_*_4.7Mo_ = 0.020 eV/atom, *E_f-_*_7.8Mo_ = 0.029 eV/atom, *E_f-_*_12.5Mo_ = 0.046 eV/atom. These trends clearly reflect a reduction in α stability and a concurrent enhancement in β phase stability with increasing Mo content. Notably, a key transition occurs in the Ti-7.8Mo alloy, where the *E_f_* of the β phase is lower than that of the α phase, marking the transition in the Ti-Mo system from α-stabilized to β-stabilized structure. However, the critical Mo content for β-phase stabilization predicted from DFT calculations (~7.0 at. %) at 0 K is slightly higher than the experimentally reported critical lower limit for β stabilization (>5.3 at. % Mo [43,44,45]). This discrepancy arises because the HCP Ti is thermodynamically favored at low temperatures, but becomes unstable with respect to BCC as temperature increases. Thus, the calculation results at 0 K are biased toward the HCP (α) phase, shifting the apparent transition point to higher Mo contents. Despite this quantitative offset, the monotonic trend that increasing Mo content enhances β-phase stability is fully consistent with experimental observations. Furthermore, as the Mo content increases, the binding energy *E_b_* of α and β phases decreases (Figure 2b), implying stronger overall bonding. Moreover, the bonding strength of the β phase surpasses that of the α phase in the Ti-7.8Mo alloy, further confirming the superior stability of the BCC structure at higher Mo concentrations. Consequently, the β phase of the Ti-12.5Mo alloy exhibits the lowest *E_f_* and *E_b_*, which are also lower than those of its α phase, making it the most favorable composition to form a single β phase under experimental conditions.

### 3.2. Mechanical Stability of Ti-Mo Binary Alloys

Based on the *E_f_* and *E_b_* analyses presented above, the Ti-4.7Mo and Ti-12.5Mo alloys were selected as representative metastable and stable systems, respectively, to evaluate the mechanical stability of the BCC structure. Typically, the mechanical stability is assessed through its elastic constants [46]. According to Born–Huang lattice dynamics theory [47,48], a mechanically stable crystal structure must possess a positive definite elastic constant matrix, meaning all its eigenvalues are positive. Consequently, the elastic constants must satisfy the specific mechanical stability criteria for a monoclinic crystal system, as given by Equations (6)–(11) [49,50]:

(6) $$C_{11}>0,\;C_{22}>0,\;C_{33}>0,C_{44}>0,C_{55}>0,C_{66}>0$$

(7) $$C_{11}+C_{22}+C_{33}+2(C_{12}+C_{13}+C_{23})>0$$

(8) $$(C_{33}C_{55}-C_{35}^{2})>0,\;(C_{44}C_{66}-C_{46}^{2})>0,\;(C_{22}+C_{33}-2C_{23})>0$$

(9) $$\left[C_{22}(C_{33}C_{55}-C_{35}^{2})+2C_{23}C_{25}C_{35}-C_{23}^{2}C_{55}-C_{25}^{2}C_{33}\right]>0$$

(10) $$\left\{2\left[\begin{array}{c} C_{15}C_{25}(C_{33}C_{12}-C_{13}C_{23})\\ +C_{15}C_{35}(C_{22}C_{13}-C_{12}C_{23})\\ +C_{25}C_{35}(C_{11}C_{23}-C_{12}C_{13}) \end{array}\right]-\left[\begin{array}{c} C_{15}^{2}(C_{22}C_{33}-C_{23}^{2})\\ +C_{25}^{2}(C_{11}C_{33}-C_{13}^{2})\\ +C_{35}^{2}(C_{22}C_{11}-C_{13}^{2})+C_{55}g_{1} \end{array}\right]\right\}>0$$

(11) $$g_{1}=C_{11}C_{22}C_{33}-C_{11}C_{23}^{2}-C_{22}C_{13}^{2}-C_{33}C_{12}^{2}+2C_{12}C_{23}C_{13}$$

where *C_ij_* (*i*, *j* = 1,…,6) are the elastic constants. In this work, the elastic constants for both alloys are determined using the stress–strain method within the framework of generalized Hooke’s law (*σ* = *C_ij_ε_j_*, *i*, *j* = 1, …, 6) [51]. For the monoclinic crystal system, there are 13 independent elastic constants, and the calculated results are listed in Table 1. The calculated elastic constants of the Ti-4.7Mo alloy violate several fundamental stability criteria, with *C*_33_ < 0, *C*_44_ < 0, *C*_55_ < 0, and *C*_66_ < 0, indicating that the β phase at this composition is mechanically unstable under multiple strain modes. Such instabilities originate from insufficient β-stabilizer content and suggest that the system is prone to phase transformation. Furthermore, the Young’s modulus of the Ti-4.7Mo alloy obtained from the *VRH* method is −44 GPa, which also confirms the mechanical instability of the BCC structure in the Ti-4.7Mo alloy. In contrast, the Ti-12.5Mo alloy satisfies all stability criteria, and its calculated Young’s modulus is 52 GPa, indicating the mechanical stability of the BCC structure at this composition. These findings are fully consistent with the formation energy trends discussed in Section 3.1.

**Figure 3:**
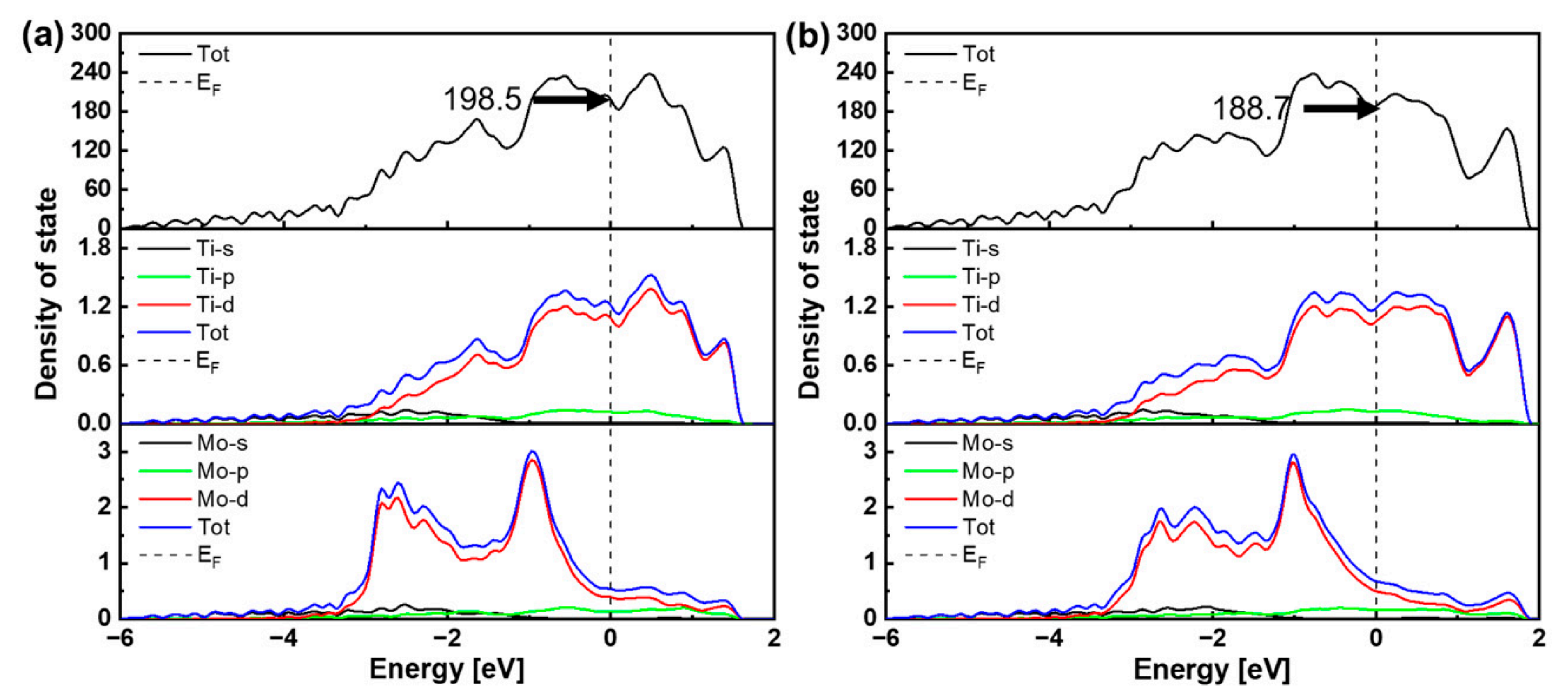
The calculated density of states (DOS) of (**a**) Ti-4.7Mo and (**b**) Ti-12.5Mo alloy.

To further understand the difference in modulus between the Ti-4.7Mo and Ti-12.5Mo alloys, the electronic density of states (DOS) of both alloys was calculated, as shown in Figure 3. The DOS profiles of the Ti-4.7Mo and Ti-12.5Mo alloys are similar in shape. In the low-energy region (~−3 eV to −1 eV), d-d orbital hybridization occurs between Ti and Mo atoms, with the characteristic peaks corresponding to bonding states between atoms, while the high-energy region (>0 eV) corresponds to anti-bonding states. Generally, the DOS at the Fermi level determines the structural stability and bonding strength. In the Ti-4.7Mo alloy, a relatively high DOS appears near the Fermi level, indicating that some electrons occupy anti-bonding (or unstable) states, which weakens the bonding strength and reduces the stability. With increasing Mo content, the Fermi level shifts closer to a pseudogap, enhancing the interatomic bonding stiffness and β-phase stability, which is manifested in higher elastic moduli [52]. Consequently, the Ti-12.5Mo alloy exhibits higher β-phase stability and modulus.

**Figure 4:**
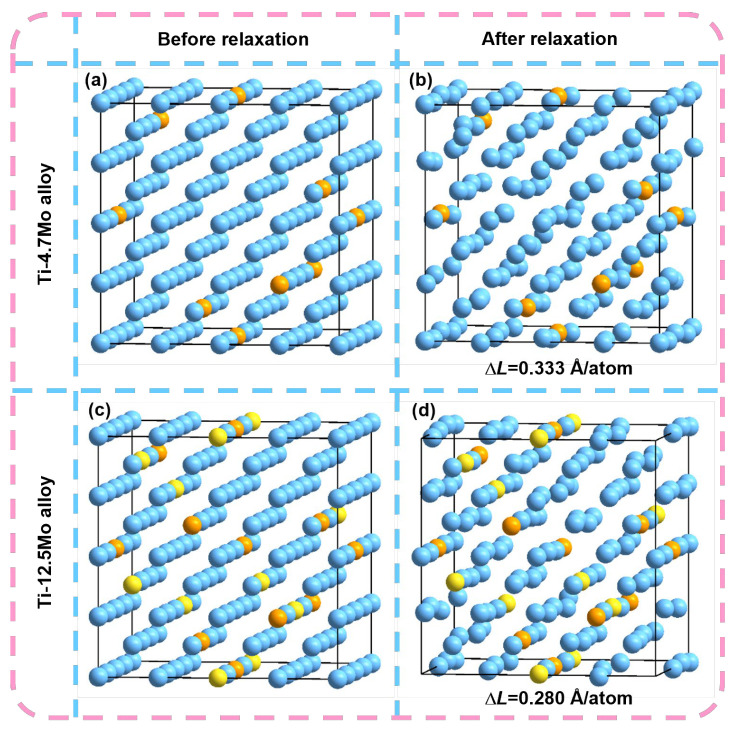
The atomic sites occupation of (**a**,**b**) Ti-4.7Mo and (**c**,**d**) Ti-12.5Mo alloys: (**a**,**c**) before and (**b**,**d**) after relaxation, with atoms colored as follows: Ti (blue), center Mo (orange), and glue Mo (yellow).

To further validate this mechanical instability from a structural perspective, we compared the atomic configurations before and after full relaxation, as shown in Figure 4. Following relaxation, the atomic site occupations of the Ti-4.7Mo and Ti-12.5Mo alloys deviate from the ideal BCC lattice. The extent of this deviation can be quantified by the lattice distortion (Δ*L*) from Equation (12) [53]:

(12) $$∆L=\frac{\left(\sum \sqrt{{\left(ax_{i}^{\prime }-x_{i}\right)}^{2}+{\left(ay_{i}^{\prime }-y_{i}\right)}^{2}+{\left(az_{i}^{\prime }-z_{i}\right)}^{2}}\right)}{n}$$

where the terms (*x_i_*, *y_i_*, *z_i_*) and (*x_i_′*, *y_i_′*, *z_i_′*) denote atomic coordinates before and after relaxation, respectively, and *n* is the total number of atoms in the cluster model. *a* is a scaling factor that normalizes the relaxed coordinates to the ideal BCC lattice, eliminating the effect of volume change. The Δ*L* values are 0.333 Å/atom for the Ti-4.7Mo alloy and 0.280 Å/atom for the Ti-12.5Mo alloy, as shown in Figure 4. Generally, a lower Δ*L* corresponds to greater BCC structural stability. The higher Δ*L* of the Ti-4.7Mo alloy further confirms that its BCC structure is unstable.

### 3.3. Deformation Behavior of Ti-Mo Binary Alloys

To clarify the connection between BCC structural stability and deformation behavior, it is critical to analyze deformation mechanisms at the atomic scale. During deformation of Ti-Mo alloys, phase transformation and twinning involve atomic-scale collapse, shear, and shuffling along specific directions, while dislocation slip proceeds via slip on close-packed planes. These processes are represented by atomic displacements. For example, the β → α″ phase transformation involves lattice elongation along {110}<110>_β_, lattice compression along {110}<100>_β_, and atomic shuffling on {110}<110>_β_ [54]; the β ⟶ ω transition involves atomic shuffling on {112}<111>_β_ [55]. Specifically, the ease of atomic displacement is related to the shear modulus *G*. The smaller the *G*, the weaker the shear resistance, which facilitates atomic displacement under stress. Thus, the propensity of each deformation mechanism can be assessed by *G* along the corresponding orientations. Since metastable Ti-Mo alloys typically deform via stress-induced α″ transition, {332}<113>_β_ twinning, and <111>_β_ dislocation slip, the orientation-dependent *G* for the metastable Ti-4.7Mo and the stable Ti-12.5Mo alloys was further calculated to investigate the underlying mechanisms of deformation behavior.

**Figure 5:**
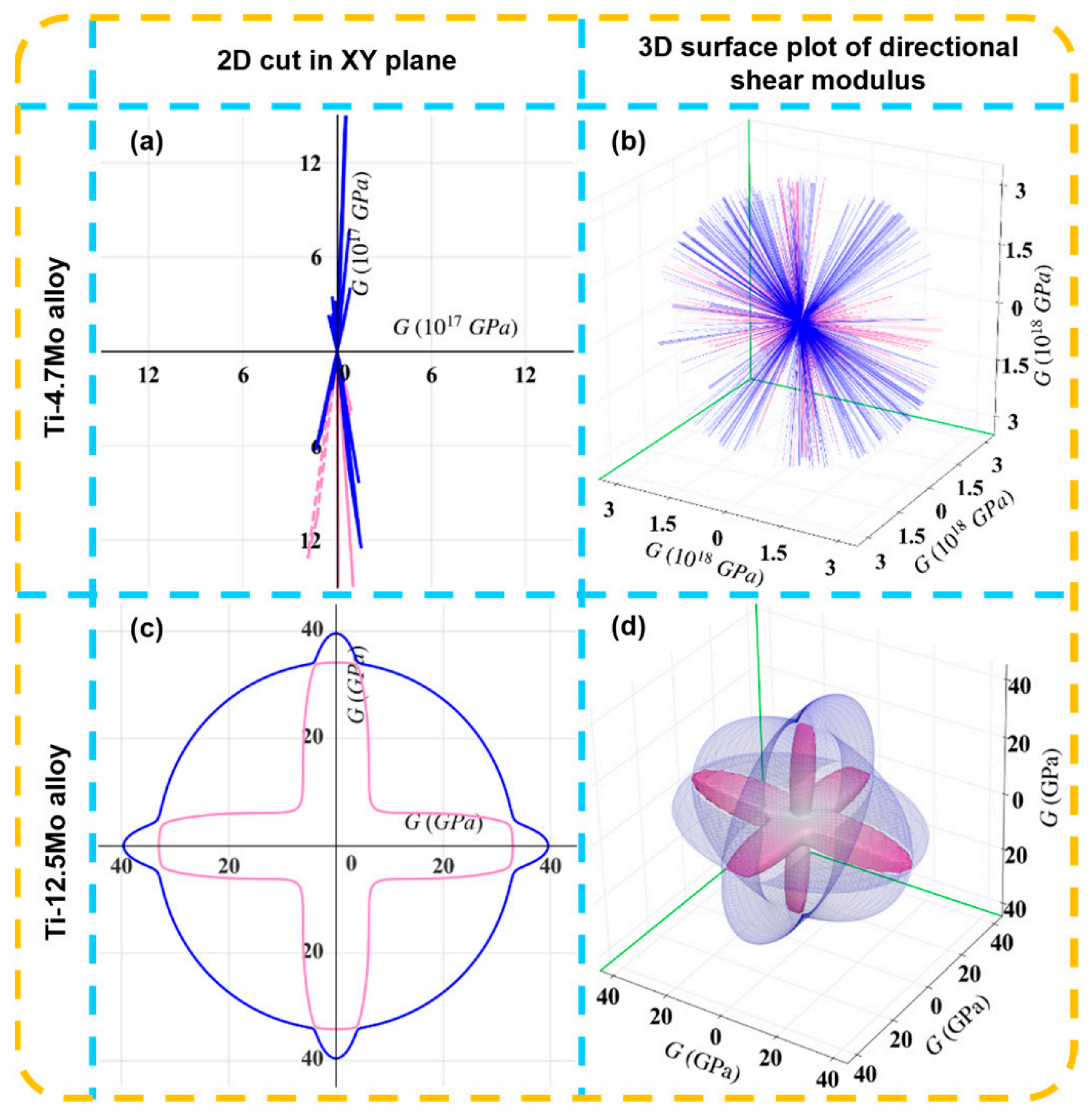
Spatial dependence of shear moduli of Ti-4.7Mo and Ti-12.5Mo alloys. (**a**,**c**): The 2D projections of shear moduli on the *XY* plane; (**b**,**d**): the 3D visualization of shear moduli. Color scheme for (**b**,**d**): *G*_max_ (blue outer surface), *G*_min_ (pink curved surface), and negative *G*_min_ (pink dotted line).

**Figure A4:**
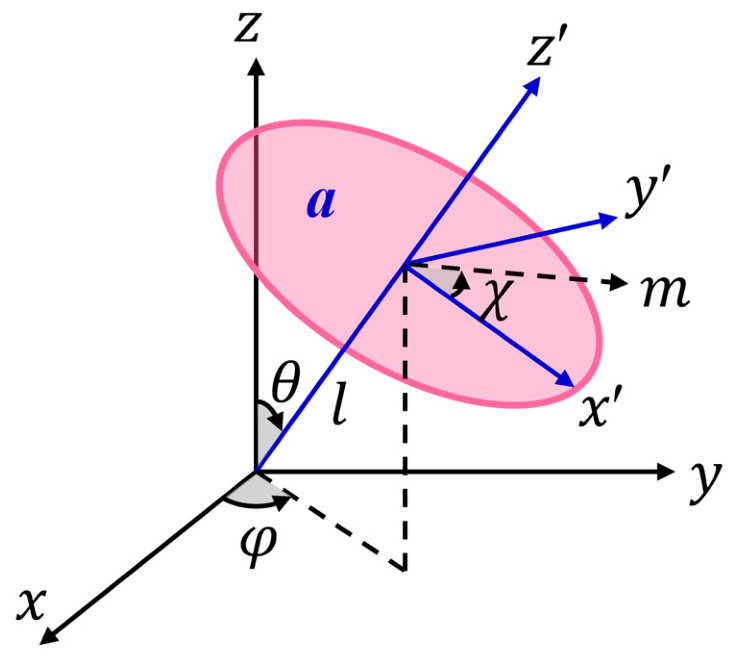
Definition of coordinate and angles. (*x*, *y*, *z*) and (*x*′, *y*′, *z*′) denote the coordinate systems of crystal structure and computational direction, respectively. *a* is the shear plane, and *z*′ represents its normal direction. *x*′ is the intersection of shear plane *a* and (*z*, *z*′) plane. *q* and *j* are polar and azimuthal angles of *z*′, respectively. *m* is the shear direction, and *χ* is the angle between *x*′ and shear direction *m*.

The orientation-dependent *G* can be obtained through a coordinate transformation [56], and the detailed formulas and transformation relationships are provided in Appendix A (Note A2) and Figure A4. Based on this, the spatial dependency of *G* for Ti-4.7Mo and Ti-12.5Mo alloys is presented in Figure 5. The three-dimensional surfaces of the orientation-dependent *G* are illustrated in Figure 5b,d. The transparent blue outer surface and the pink inner surface represent the positive *G*_max_ and *G*_min_ values, respectively, while negative values are marked by pink dotted lines. The corresponding two-dimensional projections onto the XY plane appear in Figure 5a,c. Figure 5 reveals that *G* varies with crystal orientations. For the Ti-4.7Mo alloy, owing to the instability of its BCC structure, the *G* distribution is radial and even includes negative values. In contrast, for Ti-12.5Mo with a stable BCC structure, *G* values are positive in all directions, displaying a continuous and smooth variation, with minimum and maximum values of 4 GPa and 41 GPa, respectively.

The extrema of *G* along deformation-related orientations are listed in Table 2. The Ti-4.7Mo alloy exhibits negative values of $G_{(\bar{1}12)[1\bar{1}1]}$ = −40.9 GPa and $G_{(101)[10\bar{1}]}$ = −30.4 GPa, indicating a softening effect along these crystallographic paths. As noted earlier, the β → ω transition proceeds via atomic shuffling along {112}<111>_β_, while β → α″ transformation involves lattice elongation and atomic shuffling along {110}<110>_β_, lattice compression along {110}<100>_β_. The negative values further indicate the low β phase stability in the Ti-4.7Mo alloy, making it difficult to maintain a single-phase BCC structure. Furthermore, the β → α″ transition also requires lattice compression along (101)[010]_β_ perpendicular to the lattice elongation, and the value of *G*_(101)[010]_ = 3.9 GPa is lower than those of $G_{(323)[1\bar{3}1]}$ and $G_{(110)[1\bar{1}1]}$. Consequently, the softening along ${(101)[10\bar{1}]}_{\beta }$ and the lowest positive *G* along (101)[010]_β_ promote stress-induced α″ phase transition in the Ti-4.7Mo alloy. This result is consistent with previous experimental reports that metastable BCC Ti-Mo alloys readily undergo stress-induced α″ martensitic transformations under tensile loading [13,14,15,57]. For instance, in the Ti-5.3Mo (at. %) alloy with a composition similar to that of the calculated alloy, stress-induced α″ martensitic transformation acts as the primary deformation mechanism [57].

In contrast, the Ti-12.5Mo alloy exhibits positive shear moduli across all examined orientations, with the values of $G_{(233)[\bar{3}11]}$ = 6.2 GPa, $G_{(211)[\bar{1}11]}$ = 4.8 GPa, $G_{\left(011)[01\bar{1}\right]}$ = 3.8 GPa, and $G_{\left(123)[11\bar{1}\right]}$ = 4.6 GPa. Notably, although $G_{\left(011)[01\bar{1}\right]}$ for the lattice elongation and atomic shuffling remains relatively low, *G*_(011)[100]_ for the lattice compression exhibits a high value of 36.0 GPa. Since the β → α″ transformation requires the cooperative completion of both elongation and compression steps, a low *G* along one direction alone is insufficient. The high *G*_{110}<100>_ thus suppresses α″ transformation. In the Ti-4.7Mo alloy, by contrast, *G*_(101)[010]_ is only 3.9 GPa, permitting the full β → α″ path. With the α″ transformation pathway suppressed, deformation in the Ti-12.5Mo alloy instead proceeds via twinning or dislocation slip. A comparison reveals that $G_{(233)[\bar{3}11]}$ is higher than $G_{\left(123)[11\bar{1}\right]}$. Consequently, {332}<113>_β_ twinning is not initiated preferentially. Instead, the lowest value of $G_{\left(123)[11\bar{1}\right]}$ = 4.6 GPa suggests that dislocation slip is favorable in the Ti-12.5Mo alloy. These findings match experimental observations that dislocation slip dominates the plastic deformation in stable β-type Ti-Mo alloys [44,58]. The shear modulus therefore serves as a valid mechanical descriptor to distinguish metastable from stable deformation behavior.

**Figure 6:**
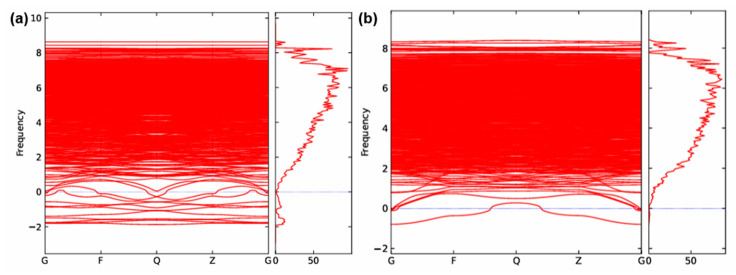
The phonon dispersions of (**a**) Ti-4.7Mo and (**b**) Ti-12.5Mo alloys.

In addition, the phonon dispersion curves of the β phase at 0 K were calculated for the Ti-4.7Mo and Ti-12.5Mo alloys. As shown in Figure 6, the Ti-4.7Mo alloy exhibits pronounced phonon softening in the dispersion curves. The corresponding density of states displays sharp peaks in the negative frequency region, indicating that the β-phase lattice at this composition is highly unstable and prone to phase transformation. In contrast, for the Ti-12.5Mo alloy, the imaginary frequencies (phonon softening) are significantly suppressed, leaving only shallow softening, and the density of states shows only a small peak. This indicates that with increasing Mo content, the lattice stiffness of the β phase increases considerably. A rigid lattice is less susceptible to large-scale shear transformation, thereby favoring dislocation slip under applied stress.

## 4. Conclusions

The present study employed FP calculations to investigate the BCC structural stability and deformation behavior of Ti-Mo binary alloys. Structural models of Ti-*x*Mo (*x* = 2.3, 4.7, 7.8, and 12.5 at. %) alloys were constructed using the cluster-plus-glue atom model to represent the CSROs in solid solutions. The β-phase stability was evaluated via *E_f_* and *E_b_*. Based on these calculations, the Ti-4.7Mo and Ti-12.5Mo alloys were selected as representative metastable and stable systems, respectively, to elucidate the deformation behavior through shear modulus calculations along key crystallographic directions. The main conclusions are as follows:

1. With increasing Mo content, the *E_f_* of the β phase decreases and that of the α phase increases, confirming that the stability of the BCC structure increases with higher Mo content. The β-phase stability transition occurs at ~7 at. % Mo, slightly above the experimentally reported threshold (>5.3 at. % Mo), likely due to the absence of temperature effects in DFT calculations.
2. In the Ti-4.7Mo alloy, $G_{(\bar{1}12)[1\bar{1}1]}$ and $G_{\left(101)[10\bar{1}\right]}$ exhibit negative values, corresponding to β → ω and β → α″ transformation paths. These negative *G* values indicate lattice instability along these directions. In contrast, the higher-Mo Ti-12.5Mo alloy shows positive shear moduli across all examined orientations, confirming the mechanical stability of its BCC structure.
3. The shear modulus can serve as an effective descriptor distinguishing between stress-induced α″ phase transformation and dislocation slip. For metastable alloys, the lowest positive value of *G*_(101)[010]_ = 3.9 GPa indicates the weak resistance to lattice compression operations required for β → α″ transformation, facilitating stress-induced α″ transformation. For stable alloys, although $G_{\left(011)[01\bar{1}\right]}$ remains relatively low, *G*_(011)[100]_ = 36 GPa is significantly higher than $G_{\left(123)[11\bar{1}\right]}$ = 4.6 GPa, thereby suppressing the α″-phase transformation and promoting dislocation slip as the dominant deformation mechanism.

## Figures and Tables

**Table 1:** FP calculated elastic constants (GPa) of Ti-Mo binary alloys.

Mo Content (at. %)	*C*_11_	*C*_12_	*C*_13_	*C*_15_	*C*_22_	*C*_23_	*C*_25_	*C*_33_	*C*_35_	*C*_44_	*C*_46_	*C*_55_	*C*_66_
Ti-4.7Mo	118.3	101.3	141.6	−2.0	73.8	163.2	53.4	−9.0	70.9	−6.7	26.0	−65.1	−64.8
Ti-12.5Mo	149.5	113.8	116.5	−1.90	122.2	109.4	4.1	114.8	10.0	34.2	−0.3	39.6	39.6

**Table 2:** Extreme values of shear moduli along the {332}<113>_β_, {112}<111>_β_, and {110}<110>_β_ directions in Ti-4.7Mo and Ti-12.5Mo alloys.

Mo Content (at. %)	*G*_{332}<113>_ (GPa)		*G*_{112}<111>_ (GPa)		*G*_{110}<110>_ (GPa)		*G*_<111>_ (GPa)	
	Max	Min	Max	Min	Max	Min	Max	Min
Ti-4.7Mo	255.6	5.9	42.0	−40.9	29.6	−30.4	94.6	6.9
Ti-12.5Mo	18.2	6.2	12.7	4.8	10.1	3.8	13.6	4.6

## Data Availability

The original contributions presented in this study are included in the article. Further inquiries can be directed to the corresponding author.

## Supplementary Materials

The following supporting information can be downloaded at: https://www.mdpi.com/article/10.3390/ma19153159/s1, Model S1: Supplementary models.Zip.

## Appendix A

Note A1: Calculation of shear moduli G_V_, G_R_ and bulk moduli B_V_, B_R_

The shear moduli *G_V_*, *G_R_*, and bulk moduli *B_V_*, *B_R_* were calculated as follows:

(A1) $$G_{V}\;=\left(\frac{1}{15}\right)[C_{11}+C_{22}+C_{33}+3(C_{44}+C_{55}+C_{66})-(C_{12}+C_{13}+C_{23})]$$

(A2) $$\begin{array}{c} G_{R}=15\{4[a(C_{11}+C_{22}+C_{12})+b(C_{11}-C_{12}-C_{23})+c(C_{15}+C_{25})+e(C_{15}-C_{25})+\\ d(C_{22}-C_{12}-C_{23}-C_{13})+f]/\Omega +3[g/\Omega +(C_{44}+C_{66})/(C_{44}C_{66}-C_{46}^{2})]{\}}^{-1} \end{array}$$

(A3) $$B_{V}=\left(\frac{1}{9}\right)[C_{11}+C_{22}+C_{33}+2(C_{12}+C_{13}+C_{23})]$$

*B_R_* = Ω [*a*(*C*_11_ + *C*_22_ − 2*C*_12_) + *b*(2*C*_12_ − 2*C*_11_ − *C*_23_) + *c*(*C*_15_ − 2*C*_25_) + 2*e*(*C*_25_ − *C*_15_) + *d*(2*C*_11_ + 2*C*_23_ − *C*_13_ − 2*C*_22_) + *f* ]^−1^ (A4)

(A5) $$a=C_{33}C_{55}-C_{35}^{2}$$

*b = C*_23_*C*_55_ − *C*_25_*C*_35_ (A6)

*c = C*_13_*C*_35_ − *C*_15_*C*_33_ (A7)

*d = C*_13_*C*_55_ − *C*_15_*C*_35_ (A8)

*e = C*_13_*C*_25_ − *C*_15_*C*_23_ (A9)

(A10) $$\begin{array}{c} f=C_{11}(C_{22}C_{55}-C_{25}^{2})-C_{12}(C_{12}C_{55}-C_{15}C_{25})+C_{15}(C_{12}C_{25}-C_{15}C_{22})\\ +\;C_{25}(C_{23}C_{35}-C_{25}C_{33}) \end{array}$$

(A11) $$g=C_{11}C_{22}C_{33}-C_{11}C_{23}^{2}-C_{22}C_{13}^{2}-C_{33}C_{12}^{2}+2C_{12}C_{23}C_{23}$$

(A12) $$\begin{array}{c} \Omega =2[C_{15}C_{25}(C_{33}C_{12}-C_{13}C_{23})+C_{15}C_{35}(C_{22}C_{13}-C_{12}C_{23})+C_{25}C_{35}(C_{11}C_{23}-C_{12}C_{13})]\\ -\;[C_{15}^{2}(C_{22}C_{33}-C_{23}^{2})+C_{25}^{2}(C_{11}C_{33}-C_{13}^{2})-C_{35}^{2}(C_{11}C_{22}-C_{12}^{2})]+gC_{55} \end{array}$$

Note A2: Calculation of orientation-dependent *G*

The orientation-dependent *G* can be obtained as follows:

(A13) $$\begin{array}{c} \frac{1}{G}\;=\;4\left[S_{11}{\left(l_{1}m_{1}\right)}^{2}\;+\;S_{22}{\left(l_{2}m_{2}\right)}^{2}\;+{\;S}_{33}{\left(l_{3}m_{3}\right)}^{2}\right]\\ +{\;S}_{44}{\left(l_{2}m_{3}\;+\;l_{3}m_{2}\right)}^{2}\;+{\;S}_{55}{\left(l_{1}m_{3\;}+{\;l}_{3}m_{1}\right)}^{2}\;+\;S_{66}{\left(l_{1}m_{2}\;+{\;l}_{2}m_{1}\right)}^{2}\\ +\;8\left[S_{12}\left(l_{1}{l_{2}m}_{1}m_{2}\right)\;+\;S_{13}\left(l_{1}{l_{3}m}_{1}m_{3}\right)\;+\;S_{23}\left(l_{2}{l_{3}m}_{2}m_{3}\right)\right]\\ +\;4l_{1}m_{1}\left[S_{14}\left(l_{2}m_{3}\;+\;l_{3}m_{2}\right)\;+{\;S}_{15}\left(l_{1}m_{3}\;+\;l_{3}m_{1}\right)\;+\;S_{16}\left(l_{1}m_{2}\;+\;l_{2}m_{1}\right)\right]\\ +\;4l_{2}m_{2}\left[S_{24}\left(l_{2}m_{3}\;+{\;l}_{3}m_{2}\right)\;+\;S_{25}\left(l_{1}m_{3}\;+\;l_{3}m_{1}\right)\;+{\;S}_{26}\left(l_{1}m_{2}\;+\;l_{2}m_{1}\right)\right]\\ +\;4l_{3}m_{3}\left[S_{34}\left(l_{2}m_{3}\;+{\;l}_{3}m_{2}\right)\;+{\;S}_{35}\left(l_{1}m_{3}\;+\;l_{3}m_{1}\right)\;+\;S_{36}\left(l_{1}m_{2}\;+{\;l}_{2}m_{1}\right)\right]\\ +\;2S_{45}\left(l_{2}m_{3}\;+\;l_{3}m_{2}\right)\left(l_{1}m_{3}\;+{\;l}_{3}m_{1}\right)\\ +\;2S_{46}\left(l_{2}m_{3}\;+\;l_{3}m_{2}\right)\left(l_{1}m_{2}\;+\;l_{2}m_{1}\right)\\ +\;2S_{56}\left(l_{1}m_{3}\;+{\;l}_{3}m_{1}\right)\left(l_{1}m_{2}\;+\;l_{2}m_{1}\right) \end{array}$$

where *S_ij_* (*i*, *j* = 1,…,6) are the components of the elastic compliance matrix (the inverse matrix of the elastic stiffness matrices *C_ij_*), *θ* and *φ* are the polar and azimuthal angles defining the orientation of the shear-plane normal *z*’, and *χ* is the angle between *x*’ and the shear direction *m*. The direction cosines *l* = (*l*_1_, *l*_2_, *l*_3_) and *m* = (*m*_1_, *m*_2_, *m*_3_) are given by:

(A14) $$\;l=\left[\begin{array}{c} \sin\theta \cos\varphi \\ \sin\theta \sin\varphi \\ \cos\varphi \end{array}\right];\;m=\left[\begin{array}{c} \cos\theta \cos\varphi \cos\chi -\sin\varphi \sin\chi \\ \cos\theta \sin\varphi \cos\chi +\cos\varphi \sin\chi \\ -\sin\theta \cos\chi \end{array}\right]$$

**Table A1:** Geometric parameters of the structural units constructed using the cluster-plus-glue-atom model for first-principles calculations.

		*a*	*b*	*c*	*α*	*β*	*γ*
Basic vector		$\sqrt{6}a_{0}$	$\sqrt{6}a_{0}$	$2\sqrt{3}a_{0}$	118.27	99.09	61.73
Atomic positions	Cluster center	0	0	0			
	1st shell of cluster	0.0625	0.1250	0.4375			
		0.2500	0.5000	0.7500			
		0.9375	0.8750	0.5625			
		0.5625	0.1250	0.9375			
		0.8750	0.7500	0.1250			
		0.1250	0.2500	0.8750			
		0.7500	0.5000	0.2500			
		0.4375	0.8750	0.0625			
	2nd shell of cluster	0.3125	0.6250	0.1875			
		0.6250	0.2500	0.3750			
		0.8125	0.6250	0.6875			
		0.1875	0.3750	0.3125			
		0.3750	0.7500	0.6250			
		0.6875	0.3750	0.8125			
	Glue atom site	0.5	0	0.5			

**Table A2:** The coordinate of Mo atoms.

Mo Content (at. %)		Coordinate
Ti-2.3Mo	3Mo	(0.625, 0.375, 0.125); (0.625, 0.875, 0.625); (0.125, 0.125, 0.875)
Ti-4.7Mo	6Mo	(0.625, 0.375, 0.125); (0.625, 0.875, 0.625); (0.125, 0.125, 0.875); (0.500, 0.500, 0.000); (0.000, 0.750, 0.250); (0.500, 0.000, 0.500)
Ti-7.8Mo	10Mo	(0.625, 0.375, 0.125); (0.625, 0.875, 0.625); (0.125, 0.125, 0.875); (0.500, 0.500, 0.000); (0.000, 0.750, 0.250); (0.500, 0.000, 0.500); (0.625, 0.625, 0.375); (0.250, 0.500, 0.500); (0.875, 0.375, 0.625); (0.500, 0.250, 0.750)
Ti-12.5Mo	16Mo	(0.625, 0.375, 0.125); (0.625, 0.875, 0.625); (0.125, 0.125, 0.875) (0.500, 0.500, 0.000); (0.000, 0.750, 0.250); (0.500, 0.000, 0.500); (0.625, 0.625, 0.375); (0.875, 0.125, 0.375); (0.875, 0.375, 0.625); (0.500, 0.250, 0.750); (0.625, 0.125, 0.875); (0.250, 0.500, 0.500); (0.500, 0.750, 0.250); (0.000, 0.500, 0.000); (0.250, 0.250, 0.250); (0.125, 0.875, 0.625)

To investigate the influence of Mo content on the stability of the BCC structure, the α-phase models were also constructed to compare the formation energies of the α and β phases. To maintain the same number of atoms as in the β-phase supercell (BCC), the α phase also adopts a 4 × 4 × 4 supercell containing a total of 128 atoms, with the number of Mo atoms set to 3, 6, 10, and 16, respectively. The corresponding α-phase models are shown in Figure A3.

The effects of the plane-wave cutoff energy (250, 300, 350, and 400 eV) and the k-point mesh (1 × 1 × 1, 2 × 2 × 2, 3 × 3 × 3, and 4 × 4 × 4) on the total energy were tested. The results indicate that with Encut = 400 eV and a 3 × 3 × 3 k-point mesh, the total energy variation is only 0.0014 eV/atom, as shown in Figure A3.
